# Purification of Mitochondrial Proteins HSP60 and ATP Synthase from Ascidian Eggs: Implications for Antibody Specificity

**DOI:** 10.1371/journal.pone.0052996

**Published:** 2013-01-10

**Authors:** Janet Chenevert, Gerard Pruliere, Hirokazu Ishii, Christian Sardet, Takahito Nishikata

**Affiliations:** 1 Université Pierre et Marie Curie and CNRS, Developmental Biology Unit UMR7009, Villefranche-sur-mer, France; 2 Frontiers of Innovative Research in Science and Technology (FIRST), Konan University, Kobe, Japan; 3 Frontier Institute for Biomolecular Engineering Research (FIBER), Konan University, Kobe, Japan; University of South Florida College of Medicine, United States of America

## Abstract

Use of antibodies is a cornerstone of biological studies and it is important to identify the recognized protein with certainty. Generally an antibody is considered specific if it labels a single band of the expected size in the tissue of interest, or has a strong affinity for the antigen produced in a heterologous system. The identity of the antibody target protein is rarely confirmed by purification and sequencing, however in many cases this may be necessary. In this study we sought to characterize the myoplasm, a mitochondria-rich domain present in eggs and segregated into tadpole muscle cells of ascidians (urochordates). The targeted proteins of two antibodies that label the myoplasm were purified using both classic immunoaffinity methods and a novel protein purification scheme based on sequential ion exchange chromatography followed by two-dimensional gel electrophoresis. Surprisingly, mass spectrometry sequencing revealed that in both cases the proteins recognized are unrelated to the original antigens. NN18, a monoclonal antibody which was raised against porcine spinal cord and recognizes the NF-M neurofilament subunit in vertebrates, in fact labels mitochondrial ATP synthase in the ascidian embryo. PMF-C13, an antibody we raised to and purified against PmMRF, which is the MyoD homolog of the ascidian *Phallusia mammillata*, in fact recognizes mitochondrial HSP60. High resolution immunolabeling on whole embryos and isolated cortices demonstrates localization to the inner mitochondrial membrane for both ATP synthase and HSP60. We discuss the general implications of our results for antibody specificity and the verification methods which can be used to determine unequivocally an antibody's target.

## Introduction

Antibody tools are of fundamental importance for learning about proteins and cellular mechanisms, and a number of recent reviews are calling for rigorous standards of antibody validation [1]–[5]. Concern about antibody specificity is increasing with the use of fluorescent protein fusions to determine protein distribution and the need to resolve discrepancies between live and fixed samples [6]. Frequently in the literature the specificity of an antibody is considered verified if it labels a single band of the expected size, or shows an affinity for the antigen produced in a heterologous system such as bacteria. Here we challenge these assumptions by directly purifying two antibody targets from the ascidian egg using both immunoaffinity methods and a novel strategy to enrich for nonabundant proteins.

**Figure 1:**
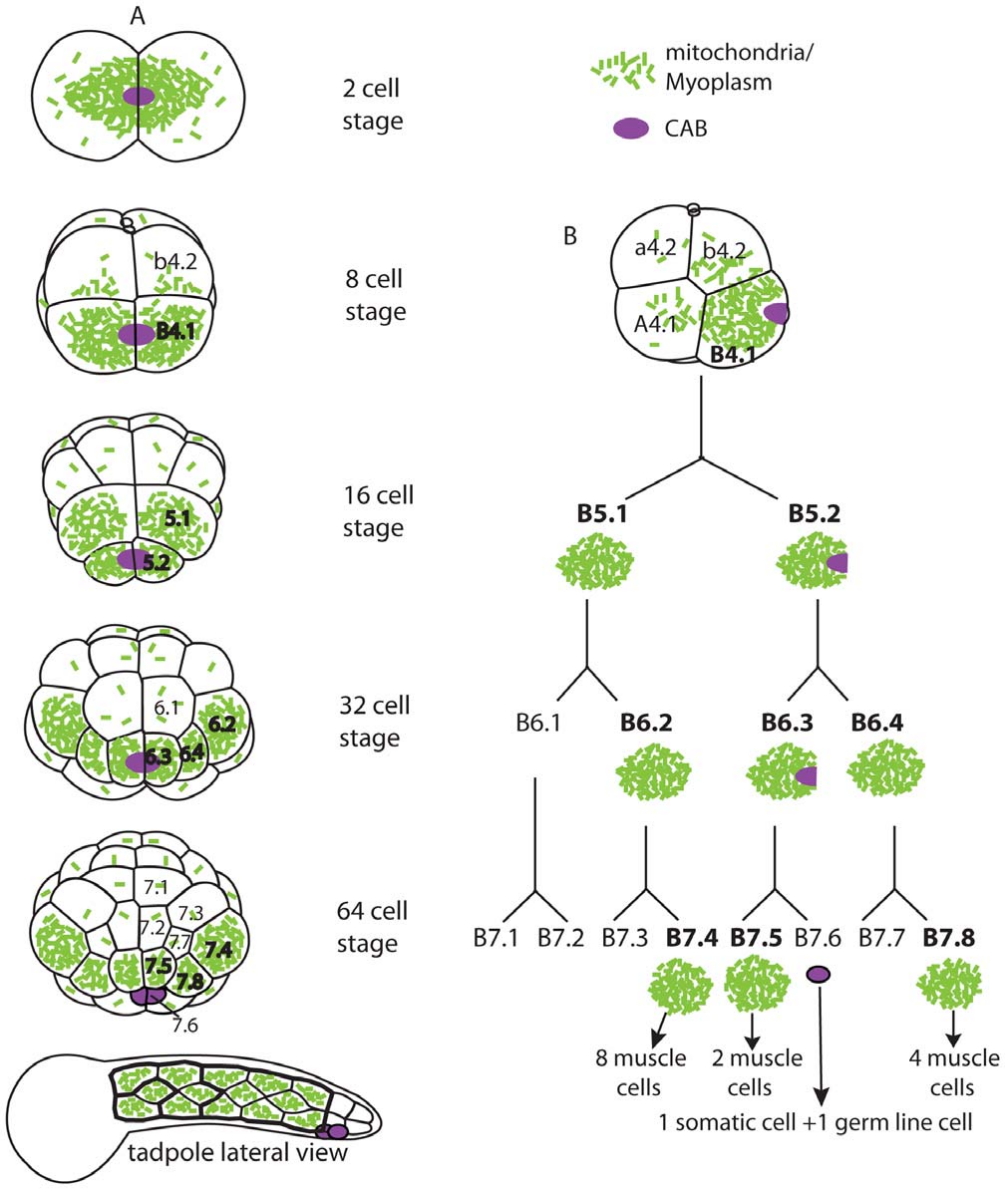
Segregation of the mitochondria-rich myoplasm into the muscle lineage of the ascidian embryo. Green speckles indicate mitochondria; the violet oval depicts the cortical domain rich in RNA, endoplasmic reticulum and germ plasm known as the CAB (for centrosome attracting body). (A) Drawings of relevant stages of the bilaterally symmetric ascidian embryo. Descendents of the B4.1 vegetal posterior blastomere are labeled. The 2 and 8 cell stages show posterior views; 16, 32, 64 cell stages are vegetal views. (B) a lateral view of the 8 cell stage and lineage of the primary muscle cells. At the 64 cell stage the bulk of the myoplasm partitions into 3 pairs of cells (B7.4, B7.5, B7.8) which give rise to the primary muscle of the tadpole tail, and the CAB domain segregates into B7.6germ line cells. Concerning myoplasm distribution during cell division, there are 3 “equal cleavages” whereby myoplasm is inherited by both daughter cells (1st cleavage, B4.1, and B5.2) and there are 6 “unequal cleavages” whereby myoplasm distributes preferentially to one daughter cell (B5.1, B6.2, B6.3, B6.4 as well as 2nd and 3rd cleavages which are not shown).

We sought to characterize the polarized mitochondria-rich domain of ascidian eggs known as the myoplasm. Ascidians are tunicates, the sister group to vertebrates [7], and their embryos develop rapidly into tadpole larvae exhibiting the typical chordate body plan [8], [9]. Over a century ago observations of the inheritance of myoplasm led biologists to propose the theory of “mosaic” or autonomous development whereby localized domains containing organelles and molecular determinants are partitioned into distinct cell lineages [10], [11]. The myoplasm is positioned as a peripheral basket in the ascidian egg, and after fertilization it moves via a series of cytoskeletal reorganizations to form a posterior crescent shape which will segregate into tail muscle [12]–[14] (Fig. 1). In living cells, the myoplasm is easily visible due to differential pigmentation, autofluorescence or via the application of vital mitochondrial dyes [10], [13], [15]–[20]. The eggs of many animals contain aggregates of tightly packed mitochondria, often associated with germ plasm, which migrate together in a mitochondrial cloud, or Balbiani body [21]–[23]. The ascidian myoplasm offers an accessible system to address questions concerning networks of mitochondria, such as their regulation, function, and how they are properly partitioned during cell division. Ascidians are amenable to an increasing battery of experimental approaches including micromanipulation, modification of gene expression or function, live imaging, genetics, proteomics [20], [24]–[28] and as we show here, biochemistry.

The most commonly used antibody tool to study ascidian myoplasm is “NN18”, a monoclonal originally made to a neurofilament preparation from pig spinal cord [29]. NN18, also called NF-160 or NF-M, reacts with the medium molecular weight (160 kDa) neurofilament subunit and labels exclusively neurofilaments in vertebrates as well as crab [30]–[33]. In the ascidian, it was found that NN18 recognizes a 58 kDa protein and strongly labels myoplasm in eggs from numerous species [13], [34]–[37]. The ascidian target of NN18 known as p58 interacts with “myoplasmin” whose sequence has features characteristic of proteins which form filaments [36], [38]. Since the myoplasm can be isolated as a unified mass [37], [39], [40] and early electron microscopy studies suggested that it contains structures resembling intermediate filaments [41]–[43], it was postulated that the ascidian target of NN18 is an intermediate filament-like protein as in vertebrates. However a more recent analysis of the *Ciona intestinalis* genome showed that ascidians lack the neurofilament (type IV) class of intermediate filaments [44], so the identity of ascidian p58 remains unclear.

Another unresolved question concerns the function of the myoplasm: it is thought to provide abundant energy for contracting myofibrils of the tadpole tail but the original proposition that it harbors molecules involved in myogenesis has not been ruled out. The myogenic determinant *Macho* RNA [45], [46] is localized to a domain rich in endoplasmic reticulum positioned just adjacent to the myoplasm, known as the CAB (see Fig. 1) [47]–[53]. Macho protein leads to activation of zygotic expression of the muscle-specific transcription factor MyoD in the muscle lineage [54]–[56]. Ascidian oocytes contain a low level of maternal mRNA encoding MyoD [57], [58] however whether a corresponding maternal MyoD protein is present in the egg or myoplasm has not been addressed.

Here we generate a polyclonal antibody against ascidian MyoD, show it labels the myoplasm, and isolate its target from ascidian eggs. We also identify with certainty the target of the standard myoplasm marker antibody NN18. Our unexpected findings have general implications for antibody “specificity” and highlight the necessity for unequivocal validation of antibody tools.

## Materials and Methods

For additional methods and details, see Methods S1.

### Animals

Adults of *Phallusia mammillata* were collected in the Mediterranean near Sete, France. All necessary permits were obtained for the described field studies from the Minister of Ecology and Sustainable Development, Marseille. The European ascidian *Phallusia* is favorable for biochemical approaches and protein purification because it produces large quantities of eggs (up to 1 ml >10^6^ eggs per hermaphroditic animal). Mature eggs are less abundant in *Ciona intestinalis* oviducts, however the *Ciona* gonad is an excellent source of maternal protein for biochemistry. This large bean-shaped organ can be up to 1 cm long and contains a virtually pure population of unfertilized eggs and immature oocytes of all stages [59]. *Ciona* adults were obtained from Roscoff marine station (Brittany, France) or through the National Bio-Resource Project NBRP of MEXT, Japan. For general ascidian protocols, fertilization and culture of embryos, see [20].

### Primary antibodies

The NN18-clone, originally generated as one of a panel of monoclonal antibodies to porcine spinal cord [29], was purchased from Sigma-Aldrich or ICN (anti-neurofilament 160 mouse monoclonal). Antibody PMF-C13 was produced by injecting a peptide present in PmMRF which is the *Phallusia* homolog of MyoD (Genbank accession HQ287931) into rabbits; immune serum was affinity purified against bacterially expressed PmMRF (Fig. S1).

### Enrichment for proteins with low affinity for ion exchange resin

In initial 2D gels loaded with total protein extract from *Phallusia* eggs or *Ciona* ovary, p63 was not abundant enough to be visible by coomassie staining, therefore a sample highly enriched for p63 was prepared by sequential ion exchange chromatography. The majority of egg proteins were eliminated by binding to a saturated DEAE column, and the flowthrough was applied to a second column on which p63 was retained. Fractions were eluted via a step salt gradient and assayed for p63 by immunoblot with PMF-C13; positive fractions were concentrated and loaded onto preparative 2D gels. See Methods S1 for details.

## Results

### NN18 and PMF-C13 antibodies recognize stable maternal proteins (p58 and p63) which distribute into the muscle lineage of ascidian embryos

**Figure 2:**
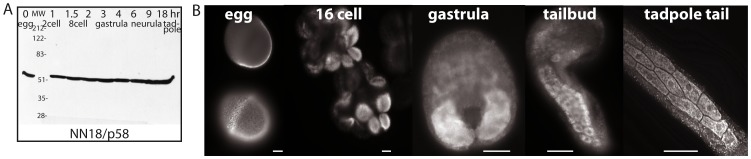
Distribution of p58 during ascidian embryonic development. (A) Immunoblot using NN18 antibody on *Phallusia mammillata* extracts prepared at the indicated stages of development; hr: hours after fertilization. (B) Examples of *Phallusia* embryos stained with NN18; egg: an equatorial view (top) or surface view (bottom) of the same unfertilized egg; 16 cell: 2 different embryos with 4 posterior cells (B5.1 and B5.2 pairs) labeled. Gastrula: posterior muscle territory is labeled. Tailbud and tadpole tail: individual mononucleate muscle cells are distinguishable. Scale bars are 20 microns in all panels.

Immunoblots using NN18 monoclonal antibody show that p58 is abundant in unfertilized eggs and remains constantly present throughout embryonic development of *Phallusia mammillata* (Fig. 2A), a pattern identical to that seen in *Ascidia ceratodes* [34] and *Ciona intestinalis* (not shown). In fixed *Phallusia* embryos, NN18 strongly labels the myoplasm and muscle lineage (Fig. 2B) [13], [49], [52], as has been observed for *Ascidia ceratodes*, *Styela clava*, *Molgula oculata*, *Ciona savigny*, and *Ciona intestinalis* [34]–[37].

**Figure 3:**
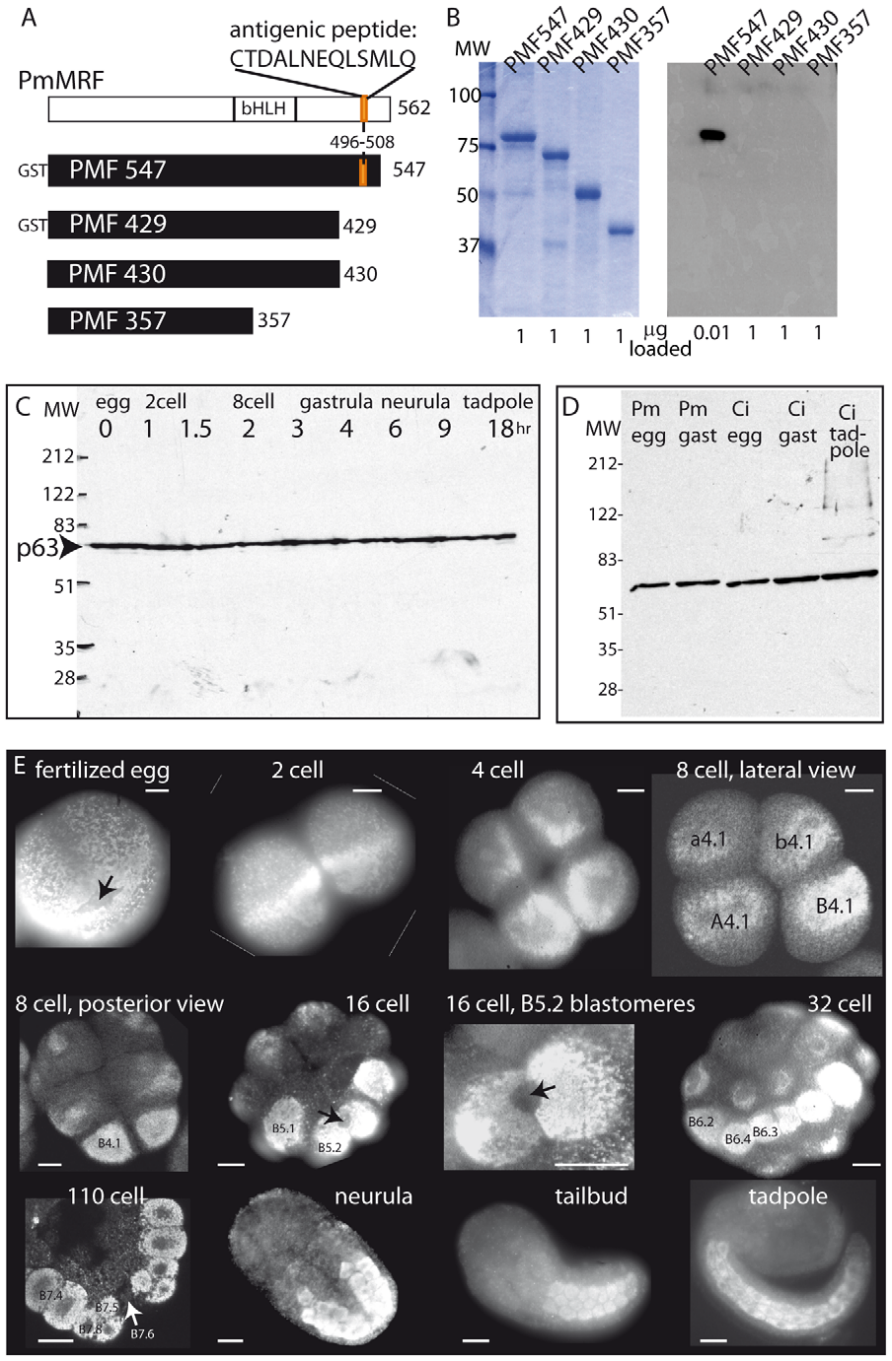
Production of antibody PMF-C13 and distribution of p63 during ascidian embryonic development. (A) Schematics of PmMRF protein (top line) and four versions produced in bacteria (black bars). bHLH: helix-loop-helix domain diagnostic of MyoD family. The number of amino acids of PmMRF present in each construct is indicated on the right. “GST” indicates that these 2 proteins also contain the 220 amino acids of Glutathione-S-Transferase. The antigenic peptide corresponding to amino acids 496–508 (colored orange) is present in “PMF547” which encodes most of PmMRF, but is absent from the others. (B) Gels containing PmMRF fusion proteins depicted in A were stained with Coomassie blue (left) or immunoblotted with antibody PMF-C13 (right). In each case 1 µg was loaded except for the PMF547 lane of the immunoblot which was loaded with much less protein (10 ng, indicated on the bottom). (C,D) Immunoblots using PMF-C13 antibody on ascidian protein extracts prepared at the indicated stages of development; hr: hours after fertilization. All samples are from *Phallusia* (Pm) except for three lanes in *D* which show PMF-C13 also recognizes p63 in *Ciona* (Ci) extracts. (E) Examples of *Phallusia* embryos stained with PMF-C13. Scale bars are 20 microns in all panels. The names in white indicate the embryonic stage and the numbers in black indicate the muscle lineage cells at the 4, 8, 16, 32 cell stages (see Fig. 1); the 64 cell stage cell names are indicated on the slightly later 110 cell embryo although B7.4 and B7.8 have already divided. Arrows indicate the cortical CAB which excludes myoplasm and thus is detectable as an unstained region surrounded by myoplasm label.

In order to address the question of maternal MyoD protein, we generated a polyclonal antibody using as antigen a 13 amino acid peptide sequence present in the *Phallusia* MyoD homolog PmMRF (Fig. 3A). This antibody, named PMF-C13, passed tests commonly cited as adequate to verify specificity for its antigen: it specifically recognizes the antigen protein PmMRF produced in bacteria, but not truncated forms of PmMRF which lack the antigenic peptide (Fig. 3B), and in ascidian extracts it strongly labels a single band of the expected size which we denote p63 (Fig. 3C,D). Like p58 (Fig. 2A), p63 is maternally provided and stably present at all embryonic stages in both *Phallusia* and *Ciona* (Fig. 3C, D). Immunofluorescence using PMF-C13 purified by affinity chromatography against PmMRF (Fig. S1) showed strong and persistent labeling of the ascidian myoplasm throughout development of *Phallusia* or *Ciona* (Fig. 3E), displaying a distribution very similar to that obtained with monoclonal NN18 (Fig. 2B). This localization pattern is surprising, since PmMRF is a myogenic transcription factor expected to be found in the nuclei of muscle cells not in association with a mitochondria-rich domain.

Thus the identity of p63, like that of p58, remained in question and has important implications for the structure and function of the ascidian myoplasm. In order to determine whether the myoplasm contains a neurofilament-like or MyoD-like protein, we set out to identify with certainty the proteins recognized by NN18 and PMF-C13, p58 and p63.

### Purification of p58 and identification as ATP synthase

**Figure 4:**
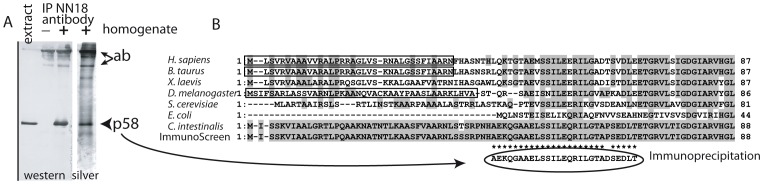
Purification of p58 by immunoprecipitation. (A) Beads to which the antibody NN18 was bound were incubated with (+) or without (−)*Ciona* oocyte homogenate and eluted proteins were subjected to immunoblot (left) or silver stain (right). The major proteins in the immunoprecipitate are p58 (arrow head) and the monoclonal antibody (arrows) which migrates as a large IgG because of the nondenaturing conditions of the gel (see text and methods). (B) N-terminal sequence of immunoprecipitated p58 (bottom line, circled) compared to ATP synthase alpha-subunit from diverse species; asterisks * indicate residues which are identical between p58 protein and *Ciona* ATP synthase alpha subunit. Immunoprecipitated p58 lacks the first 43 amino acids of ATP synthase which correspond to reported mitochondrial signal peptides (boxed). The “ImmunoScreen” line is a translation of the cDNA clones (NP_001027729) obtained by screening an expression library with NN18 antibody, which is identical to the ATP synthase gene model (KH.C10.579) annotated in the *Ciona* genome database, except in one position due to a polymorphism. See Fig. S2 for the complete alignment and database accession numbers.

The identification of p58 was accomplished by 2 independent methods: immuno-precipitation and immunoscreening. Sepharose resin coupled to NN18 antibody was incubated with a soluble fraction from *Ciona* gonad homogenate and bound proteins were separated on a non-reducing SDS-PAGE gel, so that the intact NN18 IgG would migrate at a high molecular weight well separated from the target protein. p58 was successfully immunoprecipitated as determined by immunoblot (Fig. 4A, “western”), and a duplicate lane silver-stained for total protein (Fig. 4B, right) showed that it was highly purified and abundant enough to obtain N-terminal sequence. The resulting sequence of 27 amino acid residues (circled in Fig. 4B) is identical in 26 positions to residues 44–70 of *Ciona* mitochondrial-type ATP synthase alpha-subunit. As this was an unexpected result, we also screened a *Ciona*cDNA expression library with NN18 antibody. All positive clones encoded the same protein: ATP synthase alpha-subunit “CiATPsynA” (“Immunoscreen” line, Fig. 4B). The first 43 amino acids of *Ciona* ATP synthase missing from the immunoprecipitated p58 correspond to the transit signal peptide (boxed in Fig. 4B and Fig. S2C) which targets proteins to mitochondria and is cleaved during the import process. Thus p58 protein recognized by the neurofilament NN18 antibody in the *Ciona* egg is the mature form of ATPsynthase alpha subunit. This protein along with ATP synthase beta subunit make up the intramitochondrial F1 portion of the enzyme complex which is attached to the F0 portion embedded in the mitochondrial membrane. This cross reaction is somewhat of a mystery, as there are no significant stretches of homology between ascidian ATP synthase alpha (Fig. S2B) and the initial antigenic protein, porcine NeuroFilament-M [29] (Fig. S2A), but it remains possible that they possess a short stretch of identical amino acids or a resemblance in tertiary structure.

### Purification of p63 and identification as HSP60

**Figure 5:**
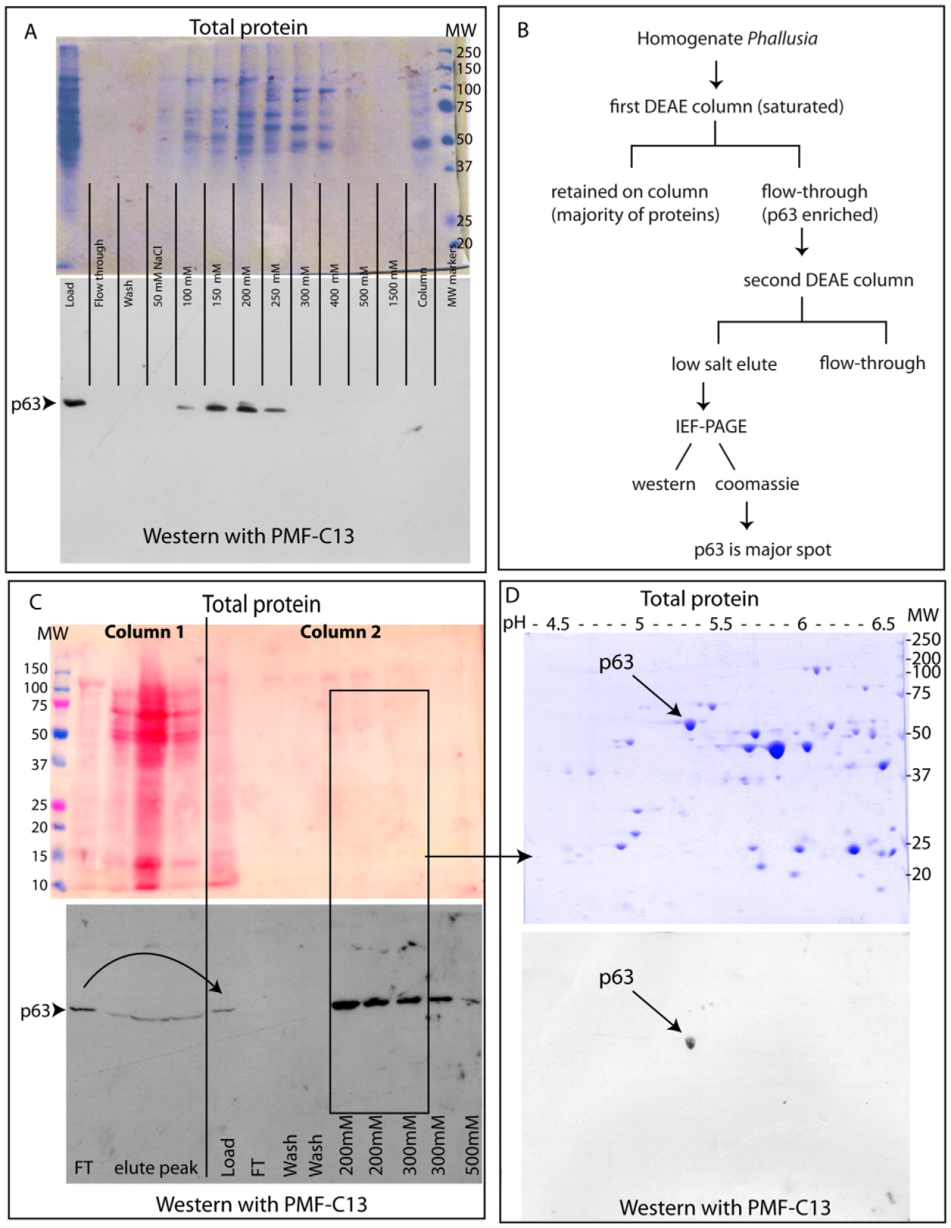
Purification of p63 using successive DEAE ion exchange columns and IEF-PAGE. In A, C and D, pairs of identical gels show distribution of p63 (western blot with PMF-C13, lower gels) compared to total protein (upper gels). (A) Total *Phallusia* egg extract prepared at low ionic strength was passed through an ion exchange column, eluted with a step salt gradient and representative fractions were analyzed; p63 (arrowhead) elutes at 100–200 mMNaCl but is not well separated from the majority of proteins. (B) Schematic of biochemical approach used to enrich p63 before 2D-electrophoresis. (C) Scale-up and sequential ion exchange columns. Column 1: The majority of proteins are retained on the overloaded column (“elute peak”) but p63 (arrowhead) passes through. The flow through (FT) from the first column (left) was loaded onto a second column as indicated by the curved arrow. Column 2: p63 elutes at 200 mM salt as in A. A fraction enriched for p63 (black rectangle) was loaded onto the duplicate 2D gels shown in D: the p63 spot (arrow) is abundant and well separated from other proteins.

Attempts at immunoprecipitation and immunoscreening with PMF-C13 were unsuccessful, and in such cases the target protein must be purified biochemically. p63 was isolated from *Phallusia* eggs by a combination of ion exchange chromatography and isoelectric focusing (Fig. 5). Initial immunoblots of 2D gels covering a range of pH revealed that the isoelectric point of p63 was approximately 5.2 but that it was not abundant enough to be distinguished as a major spot within the constellation of total egg proteins. An enrichment of p63 was obtained by a strategy of 2 sequential ion exchange columns (schematized in Fig. 5B). Initial DEAE columns indicated a relatively weak affinity for anionic exchange *DEAE* resin (Fig. 5A). A first DEAE column was intentionally overloaded with saturating amount of *Phallusia* egg extract (Fig. 5C), selecting for binding of proteins with stronger affinity for DEAE. Under these conditions the majority of proteins (over 80%) were retained, but p63 flowed through the column with a minority of proteins having weaker affinity for DEAE (Fig. 5C “column 1” FT). The flow-through from this first column which was poor in protein content but enriched for p63 was used to load a second non-saturated ion exchange column, on which p63 was retained (Fig. 5C “column 2”). The fractions eluted with 200 mM NaCl highly enriched for p63 were concentrated and loaded on two identical isoelectric focusing gels followed by PAGE.

One preparative 2D gel was stained with Coomassie blue and the twin gel was used for western blot with antibody PMF-C13 (Fig. 5D). A precise overlay of the blot and gel (see Methods S1) revealed that p63 was sufficiently abundant and pure to be excisedand subjected to mass spectrometry analysis. Peptide sequences obtained were compared to databases of the translated *Ciona* genome and subsequently to that of *Phallusia* ESTs. In both cases the strongest match was mitochondrial Heat Shock Protein 60 (HSP60) (Fig. S3A), which is a molecular chaperone of the GroEL family required for protein folding and mitochondrial activity. Ascidian HSP60 shares a similar molecular weight and isoelectric point with the antigen protein PmMRF (Fig. S3B, legend), but there is no obvious sequence or structural similarity between the two to explain the affinity of antibody PMF-C13. Nor could this target protein have been predicted: a search of *Ciona* gene models shows that 5 proteins contain a stretch of 6 consecutive amino acids in common with the antigenic peptide CTDALNEQLSMLQ, and over 200 *Ciona* proteins contain a 5 amino acid stretch, however the bona fide antibody target HSP60 is not on that list.

### ATP synthase and HSP60 are located on the inner mitochondrial membrane

Thus both the NN18 and PMF-C13 antibodies recognize not the original antigens, but instead two well conserved proteins predicted to localize in mitochondria.

**Figure 6:**
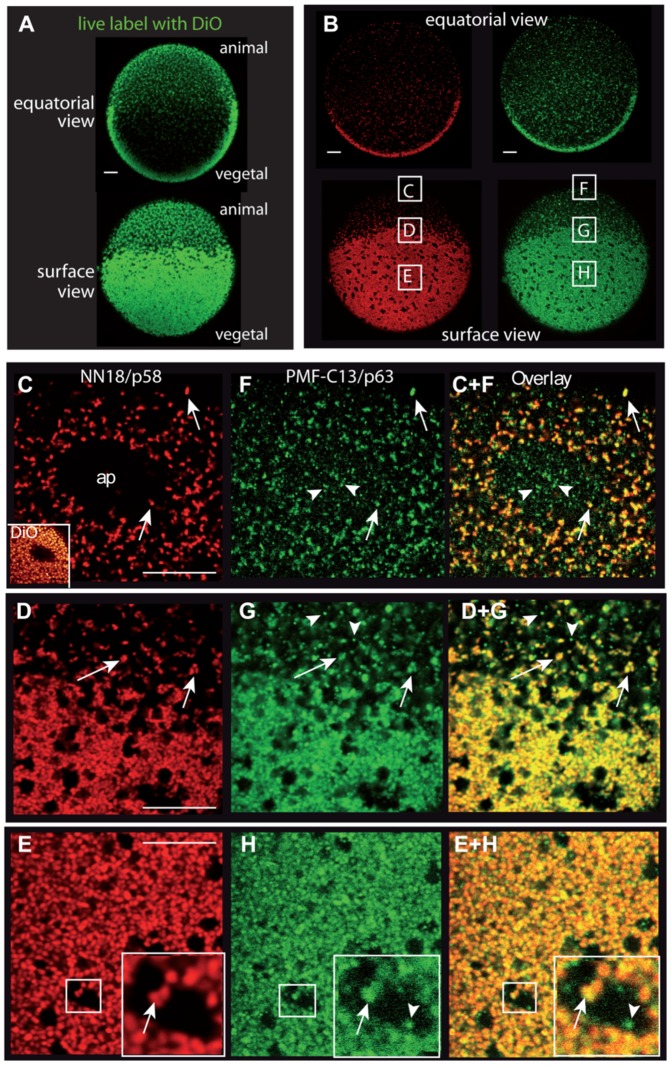
p58/ATP Synthase and p63/HSP60 localize to all mitochondria in the ascidian egg. Confocal views of *Phallusia* eggs labeled *in vivo* for mitochondria with DiOC_2_(3) (A) or fixed and double immunolabelled (B–H) with NN18 (red) or PMF-C13 (green). Eggs are oriented with the animal pole up and the vegetal hemisphere containing the myoplasm basket down. A and B show low magnification views of the center (top) or the surface (bottom) of the same egg. Boxes in B indicate positions zoomed to high magnification to detail the animal pole (C, F), the equatorial region (D,G) or the vegetal myoplasm (E,H). Yellow overlay (right panels) shows colocalization; both antibodies label individual mitochondria (arrows) and PMF-C13 also labels smaller cytoplasmic particles (arrowheads). The animal pole region (ap in C) which contains the meiotic spindle is devoid of mitochondria (as shown by live DiO labeling, inset in C). Scale bars are 10 microns in all panels.

The distribution of ATP synthase and HSP60 in whole eggs and embryos was examined by high resolution confocal microscopy (Fig. 6). In the unfertilized ascidian egg three peripheral regions can be distinguished: the vegetal layer containing the myoplasm basket rich in mitochondria (Fig. 6E and H), the animal hemisphere relatively poor in mitochondria (Fig. 6C and F), and a transition zone at the equator (Fig. 6D and G). Both NN18 (red, Fig. 6C–E) and PMF-C13 (green, Fig. 6F–H) recognize the same individual mitochondria in every region of the egg (yellow overlay, arrows in Fig. 6). Absence of signal corresponded to cell regions lacking mitochondria such as the animal pole (Fig. 6C “ap”), nuclei (Fig. S4 “n”) and the CAB (arrows in Fig. 3E, see Fig. 1). While a previous study indicated that HSP60 is found preferentially in myoplasm mitochondria [60], we observe that mitochondria in all cells of embryos and tadpoles are labelled (Fig. S4) and conclude that the myoplasm staining is due to the massive enrichment of mitochondria in this domain. In addition PMF-C13 reproducibly labeled smaller non-mitochondrial spots (arrowheads Fig. 6 F–H) distributed throughout most of the cytoplasm. NN18 labelled exclusively mitochondria in these preparations, however the use of sub-cellular fractionation followed by immunnoblotting indicates that p58 is also present in non-mitochondrial cytoplasm [61].

**Figure 7:**
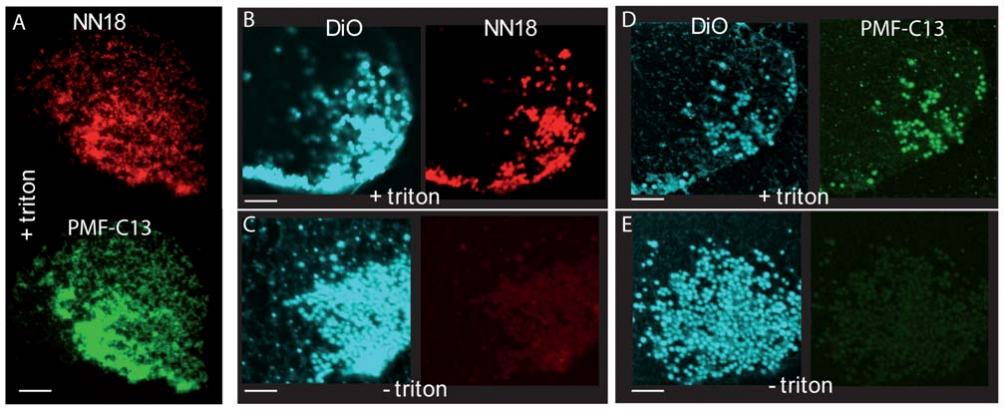
Cortices isolated from fertilized eggs and labeled with DiOC_6_(3) to show mitochondria (blue) and with antibodies NN18 (red) or PMF-C13 (green). (A,B,D) “+ triton”: cortices immunolabeled after permeabilization with detergent. (C,E) “− triton”: cortices immunolabeled in the absence of permeabilization: no antibody signal is detected. Scale bars are 5 microns.

In order to confirm mitochondrial association we used isolated cortices (Fig. 7), which are open-cell preparations of plasma membrane and adherent organelles [62], [63]. We have noted that cortices prepared from cleaving ascidian eggs retain patches of myoplasm, yielding a coverslip of semi-purified mitochondria. Such cortical preparations were first labeled with DiO(C_6_)3 to identify mitochondria, then fixed and immunolabled with antibodies. On cortices treated with triton in order to expose the inner mitochondrial membrane, NN18 and PMF-C13 antibodies label spots which colocalize with each other (Fig. 7A, B, D). In the absence of permeabilization with detergent however, antibodies only have access to the outer surface of mitochondria and NN18 and PMF-C13 do not label the isolated cortex (Fig. 7C and E, right panels). These results demonstrate that ATP synthase and HSP60 are present within mitochondria in ascidian eggs. Other subcellular localizations, such as the granules observed in Fig. 6 F–H, would not be detectable by this method however since during cortex preparation, cytosol and structures not adherent to the plasma membrane are eliminated.

## Discussion

In this study the application of biochemical methods to ascidian eggs allowed us to identify unambiguously two proteins recognized by myoplasm antibodies. Somewhat alarmingly, in both cases the antibody's target was unrelated to the original antigenic protein. The polyclonal antibody PMF-C13 showed a strong affinity for its intended target PmMRF (Figs. 3, 5, S1) and yet labels HSP60 (Figs. 5, S3). Equally unexpectedly, we find that the monoclonal antibody NN18 recognizes a protein wholly unrelated to the intermediate filament antigen, ascidian ATP synthase subunit alpha (Figs. 2, 4, S2). Thus a significant message of the present work is that use of unvalidated antibodies can lead to erroneous conclusions, such as in our case that the myoplasm is structured by intermediate filaments, or that the MyoD transcription factor localizes to mitochondria.

While the causes of these cross-reactions are unknown, their occurrence may be representative of general situations which lead to mistaken identity, some of which have been documented [4], [6], [64], [65]. For instance a secondary affinity for an abundant protein may be revealed when the presumed antigen is not present in the tissue of interest, as is the case for the NN18 antibody. The *Ciona* genome encodes 5 intermediate filament genes [44] but no homolog to the neurofilamant (typeIV) class which antibody NN18 recognizes in vertebrates. ATP synthase is one of the most abundant proteins in eggs as determined by analysis of the *Ciona* proteome (http://cipro.ibio.jp/2.5/expression profile KH.C10.579 [66]. The constant level of ATP synthase throughout embryonic development (Fig. 2A and [34] likely reflects stable maternal protein, since we find that the ATP synthase transcript is abundant in the egg and decreases during cleavage stages (Fig. S5), a pattern like that of the transcript encoding ascidian ATP translocase [67]. It is worth pointing out that since p58/ATP synthase is so stable and well-characterized, the commercially available NN18 monoclonal can be used by the ascidian community as a reliable standard to compare protein amount among samples.

In the case of the polyclonal PMF-C13, we found it recognizes HSP60 (Fig. 5, S3) even though it passed tests commonly cited to assert antigen specificity, namely that it labels a single band of the expected size and isoelectric point in the tissue of interest, and it recognizes the antigen (PmMRF) produced in a heterologous system but not truncated forms lacking the peptide sequence (Figs. 3, 5, S1). HSP60 contains 3 short sequences in common with the antigenic peptide (DAL, LNE, DALN, shaded in Fig. S3C) which could function as epitope. Indeed, since any short series of amino acids will be fortuitously present in numerous polypeptide chains, such a misleading binding of antibodies to unexpected targets may be relatively common. It is possible that antibody PMF-C13 also recognizes PmMRF in ascidian extracts, but given their similar molecular weights (Fig. S3 legend), the band corresponding to this transiently expressed transcription factor would be obscured by the stronger signal of HSP60. HSP60 is primarily a mitochondrial protein, but it is also found the cytosol and on the cell surface where it is implicated in diverse cellular functions including protein trafficking through the plasma membrane, cell signaling, apoptosis and immunological response [68]–[74]. The non-mitochondrial spots labeled by PMF-C13 (arrowheads in Fig. 6 F–H) are reminiscent of the HSP60-containing cytoplasmic granules of unknown function observed in mammalian cells [68]. HSP60 binds the translation factor YB-1 in the cytoplasm of mammalian cells [72] suggesting that the cytoplasmic HSP60 may be part of a polysome RNP complex. Interestingly in *Ciona* YB-1 binds to and regulates translation of maternal mRNAs including the localized determinants macho and PEM [75]. The ascidian and in particular the transparent *Phallusia* embryo which is favorable for live imaging [13], [20], [25] is a promising model for investigating the multiple locations and functions of HSP60 and of ATP synthase.

What methods are available for definitive validation of antibody tools and when should they be applied? It is well known that fixation conditions can radically alter the perceived distribution of a protein [6], [76]–[78] thus leading to potential misinterpretation about a protein's bona fide localization or function. To avoid fixation artifacts and to palliate the lack of antibodies it is common to infer protein localization by following fluorescently-tagged protein fusions in living cells, however an exogenous overexpressed modified protein may not faithfully reflect the distribution of the endogenous protein. Thus in general, when the distribution of a protein determined by immunolabelling is novel, unexpected, or unlike that of the GFP-tagged version, it is necessary to verify the antibody target in the species under study. In genetic model systems, homologous gene replacement or mutant collections can be employed to demonstrate that an altered coding sequence leads to the expected change in size or localization of the recognized protein. In many species and cell types one can use RNA interference or morpholino oligonucleotides to inhibit translation of a specific RNA transcript and show that there is a corresponding reduction of the candidate band or immunofluorescence signal. However proteins which are very stable and/or are provided maternally will be little affected by this type of translational inhibition. Moreover these methods are indirect: knockdown of factors involved in transcription or signaling pathways for example may also reduce the levels of their targets. Ultimately the most direct way to determine an antibody's target is to isolate it from the material of interest by the methods described in the present article: immunoprecipitation, immunoscreening of an expression library, or biochemical purification. This latter approach should become less onerous with the development of large scale proteomics, whereby researchers or commercial companies can pre-verify uncharacterized antibodies.

## Supporting Information

Figure S1 Purification of anti-peptide antibody PMF-C13 on PmMRF fusion protein. 15 ml crude rabbit serum was clarified by centrifugation and loaded onto an affigel affinity column coupled to the fusion protein encoded by the “PMF547” construct containing 547 of the 562 amino acids comprising full length PmMRF (see Fig. 3). (A) The blue line indicates amount of protein (OD260) which has passed through the BioLogic HPLC sensor during loading, washes, and acid elution. (B) Representative fractions were migrated by SDS-PAGE and stained with coomassie blue. Arrows indicate purified antibody heavy chain which has been bound to and eluted from the PmMRF fusion protein column. When an identical aliquot of antiserum was loaded onto a similar column coupled to the fusion protein encoded by the “PMF429” construct which contains 429 amino acids of PmMRF and lacks the immunogenic peptide sequence (see Fig. 3), no antibody was retained or eluted.
(TIF)

FigureS2 Amino acid sequences of proteins recognized by antibody NN18. (A) NF-M, the antigen the NN18 monoclonal antibody was originally raised against (porcine neurofilament medium, XP_001925857.1), is a protein of 934 amino acids and molecular weight 160 kD. (B) p58 is ATP synthase alpha subunit, the protein recognized by NN18 in ascidian eggs. *Ciona intestinalis* ATP synthase alpha (Genbank accession number AB071977, gene model KH.C10.579 on *Ciona* genome browser http://hoya.zool.kyoto-u.ac.jp/cgi-bin/gbrowse/kh/) [1] is a protein of 554 amino acids with calculated molecular weight 59.97 kD. *Phallusia mammillata* ATP synthase alpha (compiled by manual assembly of unpublished EST data on the bioinformatics server at Villefranche) is a protein of 556 amino acids and calculated molecular weight 60.58 kD. (C) Alignment of the complete sequences of ATP synthase alpha-subunit from *Homo sapiens* (NP_001001937), *Bos taurus* (NP_777109), *Xenopus laevis* (NP_001080447), *Drosophila melanogaster* (NP_726243), *Saccharomyces cerevisiae* (NP_009453), *Escherichia coli* (NP_418190), *Ciona intestinalis* (KH.C10.579.v1.A.SL1-1). “Cloning/NN18” is the complete sequence of the cDNA clone obtained by immunoscreening a *Ciona* expression library with NN18. “Immunoprecipitation/NN18” and “Mitochondrial Targeting Signal” are as described in Fig. 2. The ATP binding (GDRQTGKT) and ATPase (PAINVGLSVS) sequences are also indicated.
(TIF)

Figure S3 Amino acid sequences of the 2 proteins recognized by antibody PMF-C13. (A) The protein purified in Fig. 5 was subjected to tandem MS/MS sequencing, and resultant peptides compared to both *Ciona* and *Phallusia* gene model predictions. The 4 aligned peptides showed a match to the same protein HSP60, with a higher percentage of sequence identity (shaded) to *Phallusia mammillata*, the species from which p63 was isolated, than to *Ciona*. *Ciona* HSP60 (gene model KH.C6.85 on *Ciona* genome browser http://ghost.zool.kyoto-u.ac.jp/SearchGenome kh.html [1] is a protein of 573 amino acids with theoretical molecular weight 61.4 kD and isoelectric point 5.36. *Phallusia* HSP60 is a protein of 577 amino acids with theoretical molecular weight 61.9 kD and isoelectric point 5.40. The PmHSP60 sequence was compiled by manual assembly of unpublished *Phallusia* EST data on the bioinformatics server “Octopus” at Villefranche. This is a first demonstration that the well-developed *Ciona* proteomics database [2] can be used to identify proteins from the related species *Phallusia mammillata.* (B) PmMRF (MRF for Myogenic Regulatory Factor) is the *Phallusia mammillata* homolog of MyoD; the 13 amino acid C-terminal peptide used as antigen is highlighted. PmMRF (accession number HQ287931) is a protein of 562 amino acids with theoretical molecular weight 62.1 kD and isoelectric point 5.02. (C) The short highlighted sequences are regions of ascidian HSP60 with at least 3 consecutive amino acids identical to the immunogenic peptide.
(TIF)

Figure S4 Posterior region of *Phallusia* embryos stained with antibodies NN18 (red) and PMF-C13 (green). (A) Muscle precursor cells in 64 cell stage embryo, 4 hours after fertilization. n: nucleus. (B) muscle cells in a portion of the tadpole tail, which is formed 18 hours after fertilization. Non-myoplasm mitochondria which are outside of muscle lineage (arrows) are labeled as well as myoplasm mitochondria. Scale bars are 10 microns.
(TIF)

Figure S5 Spatial and temporal expression pattern of CiATPsynthase mRNA. (A) RT-PCR using primers specific for ATP synthase alpha or for tubulin control. (B) Whole mount in situ hybridization of embryos fixed at the indicated times in development. CiATPsyn transcript accumulates during oogenesis and is evenly distributed in the unfertilized egg. During cleavage stages the amount of CiATPsyn message decreases and is hardly detectable in neurula and later stages. Scale bars are 50 microns.
(TIF)

Methods S1 (DOCX)
