# Supplementary figures and images for: Purification of Mitochondrial Proteins HSP60 and ATP Synthase from Ascidian Eggs: Implications for Antibody Specificity
Source: PLoS One. 2013 Jan 10;8(1):e52996. doi: 10.1371/journal.pone.0052996 (PMC3542361; doi:10.1371/journal.pone.0052996)

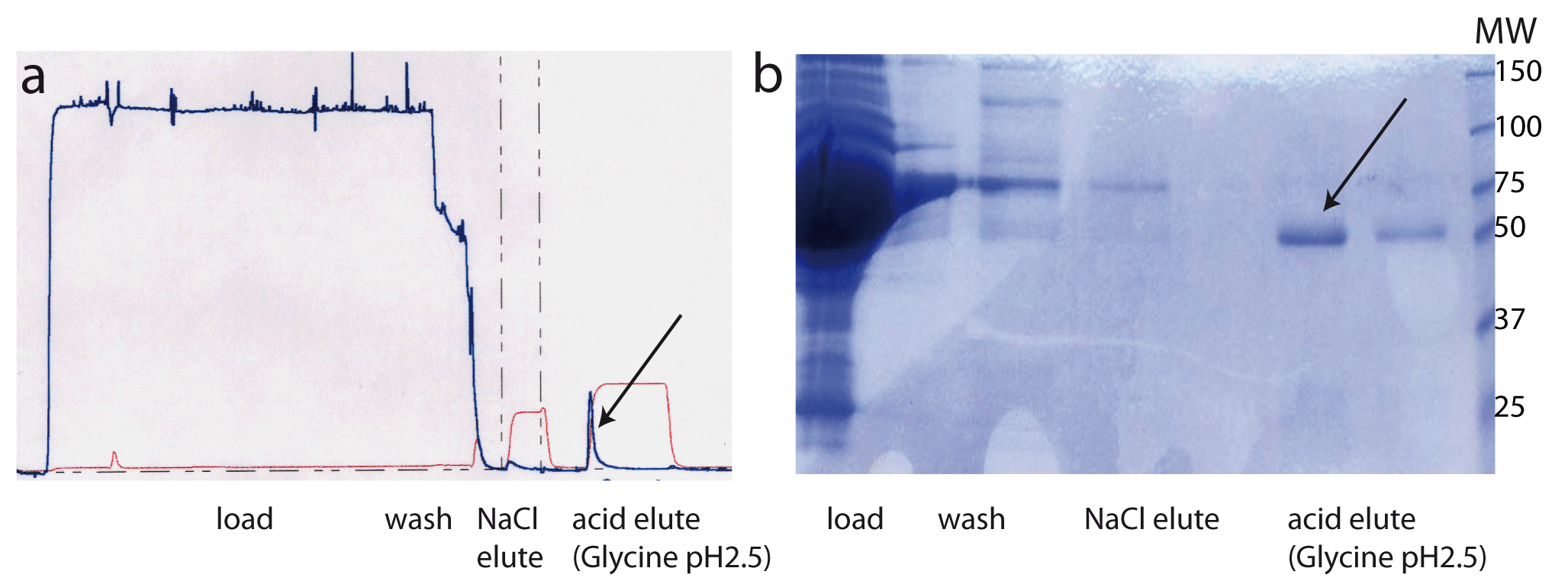

Supplement: Figure S1 — Purification of anti-peptide antibody PMF-C13 on PmMRF fusion protein. 15 ml crude rabbit serum was clarified by centrifugation and loaded onto an affigel affinity column coupled to the fusion protein encoded by the “PMF547” construct containing 547 of the 562 amino acids comprising full length PmMRF (see Fig. 3). (A) The blue line indicates amount of protein (OD260) which has passed through the BioLogic HPLC sensor during loading, washes, and acid elution. (B) Representative fractions were migrated by SDS-PAGE and stained with coomassie blue. Arrows indicate purified antibody heavy chain which has been bound to and eluted from the PmMRF fusion protein column. When an identical aliquot of antiserum was loaded onto a similar column coupled to the fusion protein encoded by the “PMF429” construct which contains 429 amino acids of PmMRF and lacks the immunogenic peptide sequence (see Fig. 3), no antibody was retained or eluted. (TIF) [file pone.0052996.s001.tif]

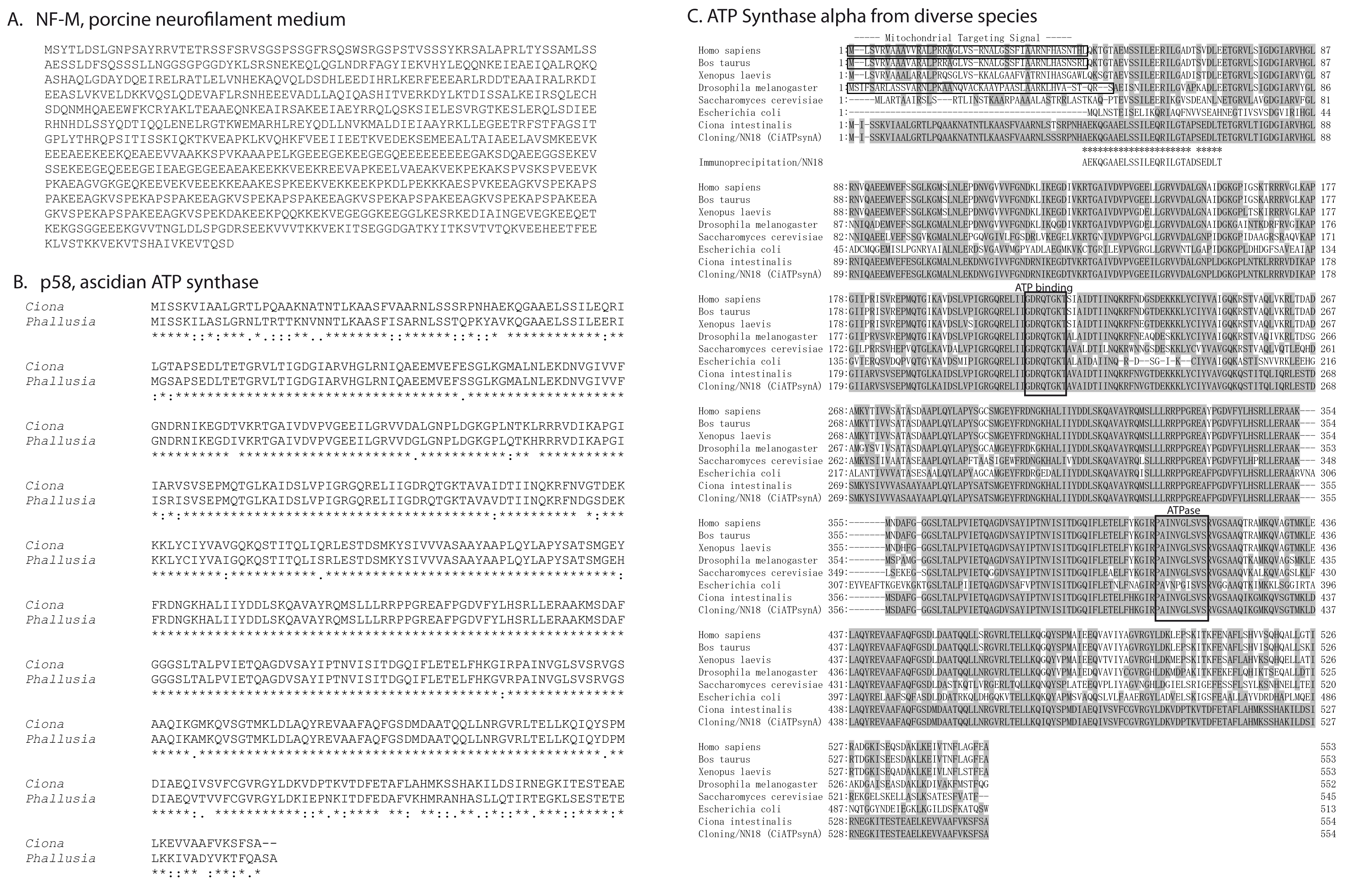

Supplement: FigureS2 — Amino acid sequences of proteins recognized by antibody NN18. (A) NF-M, the antigen the NN18 monoclonal antibody was originally raised against (porcine neurofilament medium, XP_001925857.1), is a protein of 934 amino acids and molecular weight 160 kD. (B) p58 is ATP synthase alpha subunit, the protein recognized by NN18 in ascidian eggs. Ciona intestinalis ATP synthase alpha (Genbank accession number AB071977, gene model KH.C10.579 on Ciona genome browser http://hoya.zool.kyoto-u.ac.jp/cgi-bin/gbrowse/kh/) [1] is a protein of 554 amino acids with calculated molecular weight 59.97 kD. Phallusia mammillata ATP synthase alpha (compiled by manual assembly of unpublished EST data on the bioinformatics server at Villefranche) is a protein of 556 amino acids and calculated molecular weight 60.58 kD. (C) Alignment of the complete sequences of ATP synthase alpha-subunit from Homo sapiens (NP_001001937), Bos taurus (NP_777109), Xenopus laevis (NP_001080447), Drosophila melanogaster (NP_726243), Saccharomyces cerevisiae (NP_009453), Escherichia coli (NP_418190), Ciona intestinalis (KH.C10.579.v1.A.SL1-1). “Cloning/NN18” is the complete sequence of the cDNA clone obtained by immunoscreening a Ciona expression library with NN18. “Immunoprecipitation/NN18” and “Mitochondrial Targeting Signal” are as described in Fig. 2. The ATP binding (GDRQTGKT) and ATPase (PAINVGLSVS) sequences are also indicated. (TIF) [file pone.0052996.s002.tif]

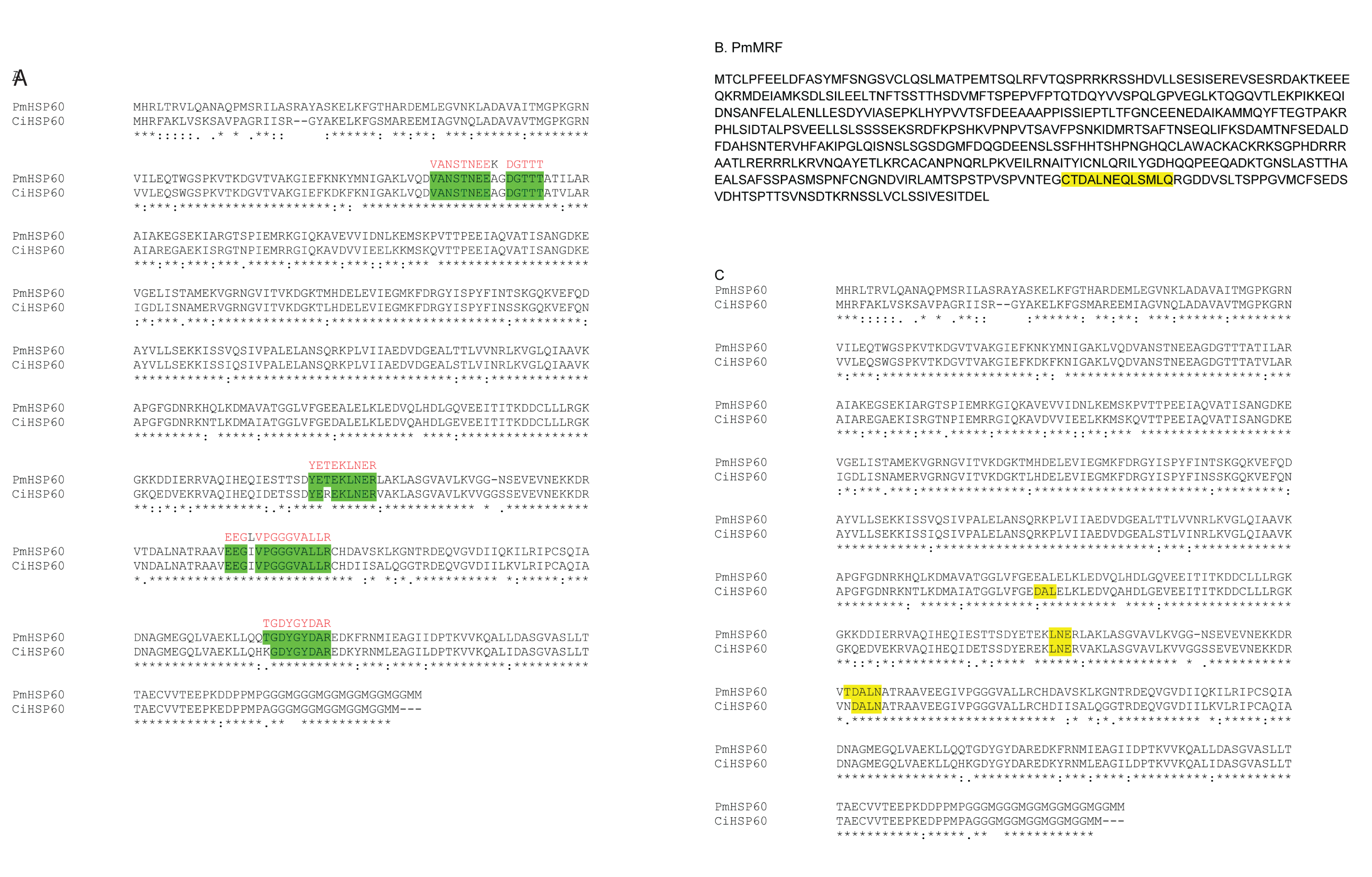

Supplement: Figure S3 — Amino acid sequences of the 2 proteins recognized by antibody PMF-C13. (A) The protein purified in Fig. 5 was subjected to tandem MS/MS sequencing, and resultant peptides compared to both Ciona and Phallusia gene model predictions. The 4 aligned peptides showed a match to the same protein HSP60, with a higher percentage of sequence identity (shaded) to Phallusia mammillata, the species from which p63 was isolated, than to Ciona. Ciona HSP60 (gene model KH.C6.85 on Ciona genome browser http://ghost.zool.kyoto-u.ac.jp/SearchGenome kh.html [1] is a protein of 573 amino acids with theoretical molecular weight 61.4 kD and isoelectric point 5.36. Phallusia HSP60 is a protein of 577 amino acids with theoretical molecular weight 61.9 kD and isoelectric point 5.40. The PmHSP60 sequence was compiled by manual assembly of unpublished Phallusia EST data on the bioinformatics server “Octopus” at Villefranche. This is a first demonstration that the well-developed Ciona proteomics database [2] can be used to identify proteins from the related species Phallusia mammillata. (B) PmMRF (MRF for Myogenic Regulatory Factor) is the Phallusia mammillata homolog of MyoD; the 13 amino acid C-terminal peptide used as antigen is highlighted. PmMRF (accession number HQ287931) is a protein of 562 amino acids with theoretical molecular weight 62.1 kD and isoelectric point 5.02. (C) The short highlighted sequences are regions of ascidian HSP60 with at least 3 consecutive amino acids identical to the immunogenic peptide. (TIF) [file pone.0052996.s003.tif]

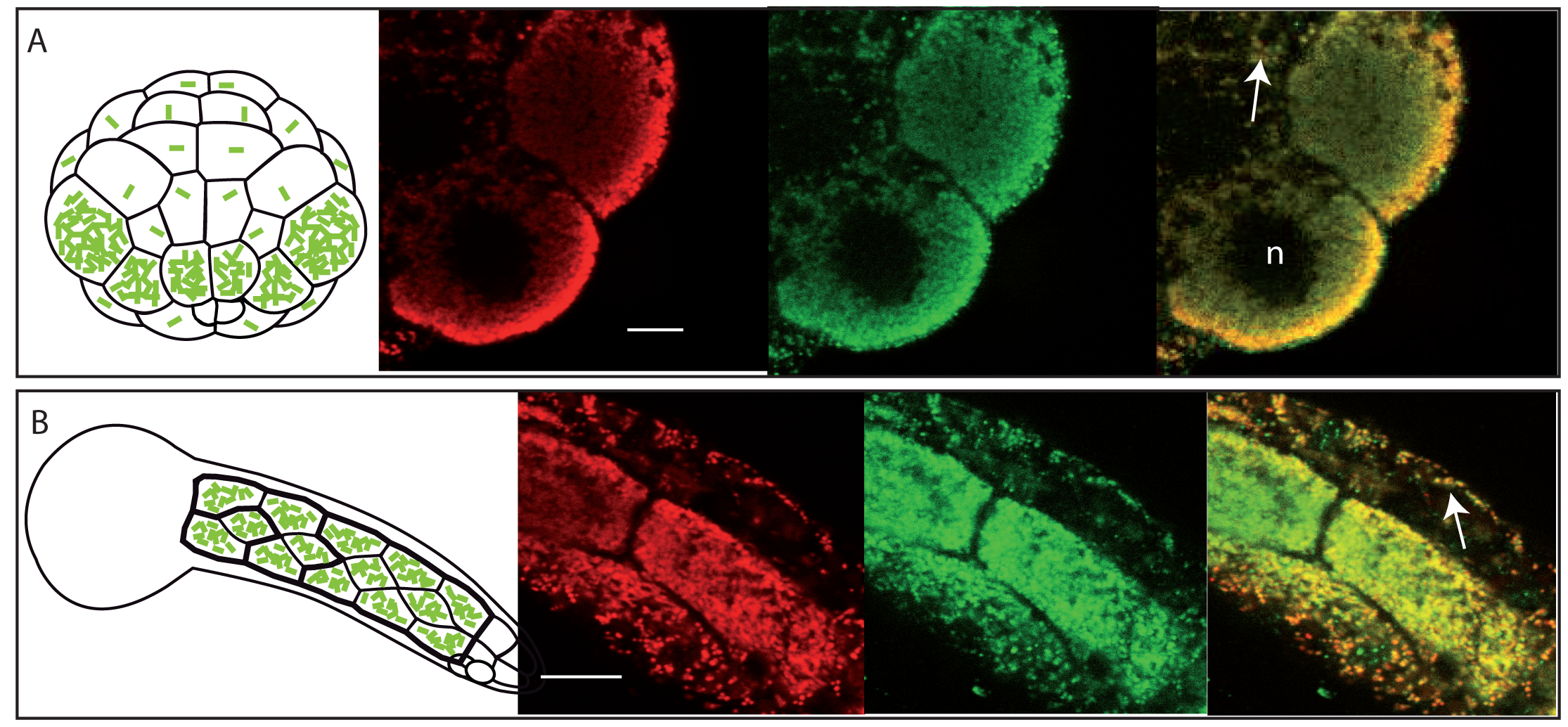

Supplement: Figure S4 — Posterior region of Phallusia embryos stained with antibodies NN18 (red) and PMF-C13 (green). (A) Muscle precursor cells in 64 cell stage embryo, 4 hours after fertilization. n: nucleus. (B) muscle cells in a portion of the tadpole tail, which is formed 18 hours after fertilization. Non-myoplasm mitochondria which are outside of muscle lineage (arrows) are labeled as well as myoplasm mitochondria. Scale bars are 10 microns. (TIF) [file pone.0052996.s004.tif]

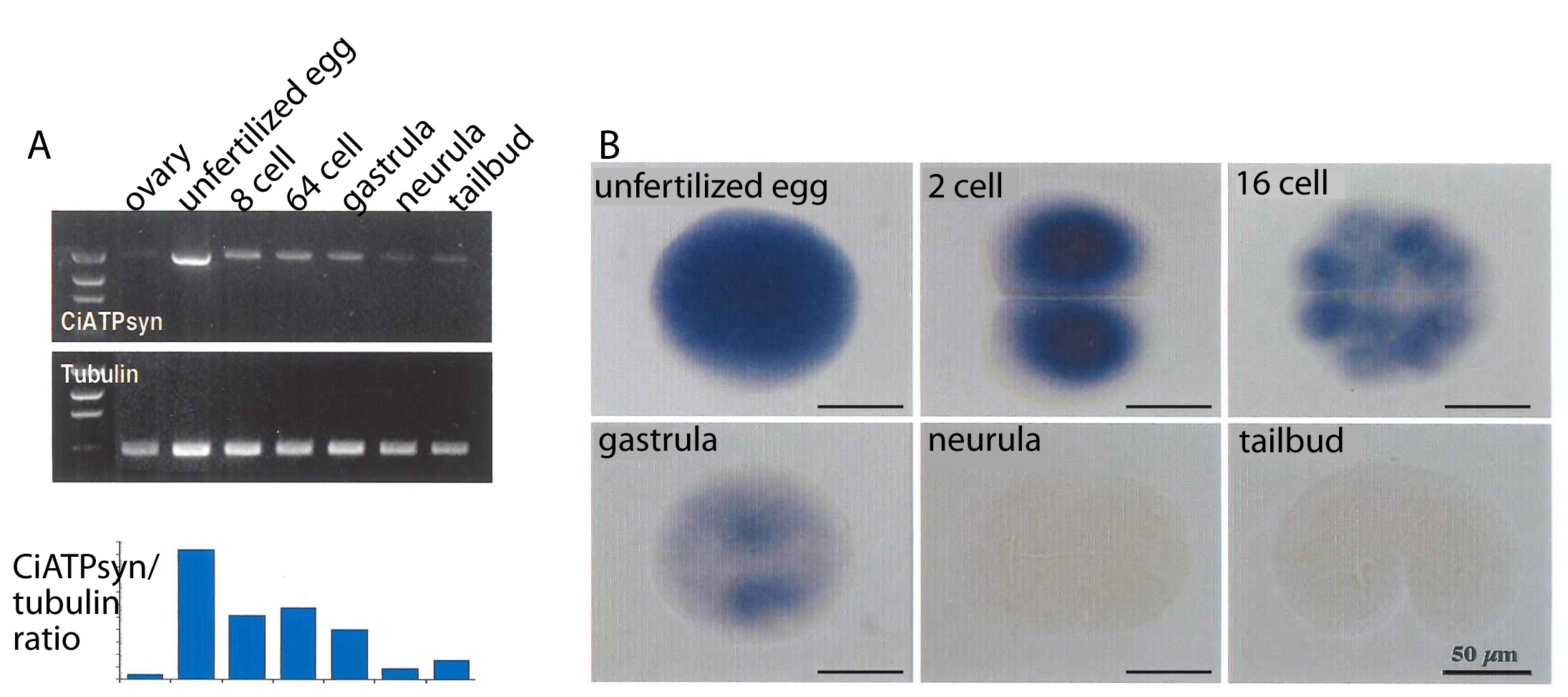

Supplement: Figure S5 — Spatial and temporal expression pattern of CiATPsynthase mRNA. (A) RT-PCR using primers specific for ATP synthase alpha or for tubulin control. (B) Whole mount in situ hybridization of embryos fixed at the indicated times in development. CiATPsyn transcript accumulates during oogenesis and is evenly distributed in the unfertilized egg. During cleavage stages the amount of CiATPsyn message decreases and is hardly detectable in neurula and later stages. Scale bars are 50 microns. (TIF) [file pone.0052996.s005.tif]
