# Supplementary material for: Purification of Mitochondrial Proteins HSP60 and ATP Synthase from Ascidian Eggs: Implications for Antibody Specificity
Source: PLoS One. 2013 Jan 10;8(1):e52996. doi: 10.1371/journal.pone.0052996 (PMC3542361; doi:10.1371/journal.pone.0052996)
Supplement: Methods S1 — (DOCX) [file pone.0052996.s006.docx]

**Supplementary Methods**

**Preparation of protein extracts from ascidian eggs and embryos**. For developmental immunoblots as in Figs. 2 and 3, samples of dechorionated eggs or embryos are collected in an eppendorf tube and centrifuged briefly. The sea water is rapidly removed by aspiration, and the dry embryo pellet is frozen in liquid nitrogen. 1 µl packed pellet contains approximately 1000 eggs. Quantitative measurements indicate that 1 *Phallusia mammillata* egg contains 50-100 ng protein. The pellet is lysed in Laemmli sample buffer by vortexing or pipetting, heated to 95°C for 5 minutes, and centrifuged briefly before loading. Standardized amounts are obtained by resuspending each pellet in 10 times its volume (for example 100 µl Laemmli for 10 µl packed eggs) and loading 5 µl, which corresponds to approximately 500 eggs or 50 µg protein per lane.

**Production and purification of antibody PMF-C13.** *Phallusia* MyoD (“PmMRF”; Genbank accession HQ287931) was first cloned by screening a neurula stage cDNA library with a probe generated by PCR using degenerate primers flanking the conserved helix-loop-helix domain (indicated in Fig. 3). A peptide predicted to be antigenic corresponding to amino acids 496-508 of PmMRF (CTDALNEQLSMLQ) was synthesized and injected into rabbits by CovalAb (France). Crude rabbit antisera were clarified by ultracentrifugation at 30,000 rpm for 45 min, diluted 5X with cold 10mM Tris pH 7.5, passed through a 22 µm filter, and purified against the PmMRF fusion protein encoded by the “pGexEco” construct (Figs. 3, S1) which consisted of nearly the entire coding sequence of PmMRF (547 of 562 amino acids) cloned into pGEX2 vector (Pharmacia Biotech), produced in bacteria and immobilized on Affi-Gel resin (BioRad). The affinity columns were run at 4°C using either the BioLogic Chromatography Workstation (BioRad) or peristaltic pumps. The antibody was eluted with 0.1 M glycine pH 2.5 (Fig. S1), neutralized with 1/10 volume 1M Tris pH8, dialyzed against PBS, concentrated if necessary with Centricon units (Millipore), and frozen at -80°C at a concentration > 1 mg/ml. Western blots demonstrated that antibody PMF-C13 recognizes specifically full length PmMRF, but not shortened forms of PmMRF protein which lack the antigenic peptide (Fig. 3).

**Immunolabelling.** Dechorionation, fertilization and culture of *Ciona* and *Phallusia* eggs were as described (Sardet et al 2011). To observe the myoplasm in living eggs (Fig. 6A) the samples were treated with 1 µg/µl DiOC_2_(3), a mitochondrial dye which emits fluorescence in response to increasing membrane potential. For antibody labelling, eggs and embryos were fixed in 4% paraformaldehyde, 0.5M NaCl in PBS for 2 hours at room temperature or overnight at 4°C, then washed twice in PBS, once in 50% ethanol, once in 90% ethanol, and stored at –20°C in ethanol. Embryos fixed and stored directly in –20°C methanol gave similar results. Immunolabelling was as described (Patalano et al 2006): fixed samples were rehydrated in PBS + 0.1% Tween 20 (PBSTw) and blocked for 30 minutes in 1% Bovine Serum Albumin in PBSTw. The volume was reduced to 50 µl, and primary antibodies NN18 or PMF-C13 were added at a dilution of 1:200. The embryos were incubated with primary antibodies under gentle agitation or occasional resuspension for 2 hours at 25°C or overnight at 4°C, washed three times with PBSTw, and incubated with fluorescent secondary antibody (FITC-conjugated anti-rabbit or Texas Red-conjugated anti-mouse (Jackson Labs) at a final dilution of 1/200 for 2 hours at room temperature. The samples were then washed twice with PBS-Tw, incubated with 5 µg/ml Hoechst 32222 (Merck) in PBS-Tw for 10 minutes, and washed twice more with PBSTw. They were mounted in a drop of Citifluor AF1 mounting medium (Citifluor Ltd London) and observed using a Zeiss Axiovert or Leica SP2 confocal microscope. Since the myoplasm displays a low level of natural autofluorescence (which decreases upon fixation) a control sample incubated with secondary but not primary antibody was always included for comparison.

**Immunoprecipitation.** *Ciona intestinalis* gonads were collected and frozen in liquid nitrogen, then pulverized with a mortar and a pestle without thawing and stored in aliquots at -80°C. Just before immunoprecipitation, frozen gonad material was lysed in 10 volumes of ice cold homogenization buffer (20 mM HEPES pH 7.5, 150 mM NaCl, 1 mM EDTA , 1% Triton X-100) containing 10 µg/ml leupeptin and 1 mM PMSF or protease inhibitor cocktail (P8340, Sigma-Aldrich) and homogenized for 10 strokes with a Potter-Elvehjem glass homogenizer fitted with a Teflon pestle. The homogenate was centrifuged at 5000 x g for 10 min and the supernatant was used immediately.

NN18 antibody was bound to protein-G sepharose resin (Amersham Pharmacia Biotech) by incubating at 4°C for 1 hr. The gonad homogenate (soluble fraction) was pre-treated with protein-G sepharose for 1 hr at 4°C in order to reduce non-specific binding, and then incubated together with the NN18 bound protein-G sepharose for 24 hours at 4°C with gentle rocking. The resin pellet containing immunoprecipitated proteins was washed extensively with PBS, and soaked with SDS-sample buffer not containing 2-mercaptoethanol (0.0625 M Tris-HCl pH 6.8, 10% glycerol, 0.5 µM PMSF, 2.3% SDS). Nondenaturing conditions were used during sample preparation and electrophoresis in order to separate the targeted protein (58 KD) from immunoglobulin heavy chain (55 kD) which also eluted from the sepharose.

The products of immunoprecipitation were loaded onto SDS-PAGE gels without mercaptoethanol which were either silver stained or blotted onto PVDF membrane (Immobilon; Millipore). The band corresponding to p58 was excised from a PVDF membrane stained with Coomasie Brilliant Blue and sequenced by the N terminal Edman degradation method using a Model Procise 491 (Applied Biosystems) machine.

**Immunoscreening of cDNA library:** Starting with RNA extracted from *Ciona intestinalis* unfertilized eggs, an oligo-d(T)-primed cDNA library was constructed in Uni-ZAP XR and screened according to the manufacturer’s method (Stratagene). About 50,000 plaques were plated, induced and probed with the NN18 monoclonal antibody, yielding 10 positive cDNA clones.

**In situ Hybridization.** Fixation of embryos for detection of ATP synthase transcript and in situ hybridization RNA probes (Fig. S5) were performed as in Meedel et al, 2007.

**Ion exchange chromatography.** Initial small scale prep (Fig. 4A): To prepare *Phallusia* protein extract, 800 µl of packed eggs was lysed in 8 mls “lysis buffer” (20mM Hepes pH7, 0.5mM EDTA, 0.5mM DTT, 0.1% NP40) and clarified by centrifugation at 20,000 rpm for 15 minutes at 4°C. The supernatant was incubated for 3 hours with 2ml DEAE cellulose (Whatman) pre-equilibrated in lysis buffer pH7. The DEAE column was washed with 10 volumes of “column buffer” (20 mM Tris pH7, 0.5mM EDTA, 0.5mM DTT, 10 % glycerol, 10 mM PMSF) in order to eliminate cell debris and unbound protein, and eluted with a step gradient of 0 to 500 mM NaCl in column buffer, followed by a final step at 1.5 M NaCl to ensure that all proteins were released. Fractions were analyzed by SDS-PAGE followed by coomassie stain or immunoblot to determine the amount of total protein or p63, respectively. Fraction 2 containing the peak of eluted protein was diluted 20 fold in column buffer pH7 in order to decrease the salt concentration and loaded for 2 hrs at 4°C onto 1.5ml DEAE cellulose. This column was washed with 10 volumes of column buffer and eluted with a step gradient of 50 to 1500 mM NaCl, and representative fractions were analyzed.

Scaled-up prep for purification (Fig. 4C,D): 5 ml *Phallusia* dechorionated eggs (approximately 500 µg total protein) was resuspended in 45 ml lysis buffer, pulverized with 10 strokes of a 40 ml Dounce tissue grinder (Wheaton) and centrifuged at 20K for 30 minutes at 4°C. 40 mls supernatant was mixed overnight at 4° with 4 ml DEAE cellulose pre-equilibrated in lysis buffer. The column was washed twice with 30 ml column buffer and eluted with high salt (1.5ml NaCl in column buffer). The flowthrough containing the protein of interest (p63) was centrifuged 1 hour at 100,000g. This supernatant was diluted 2 fold giving a final volume of 90 mls, which was incubated with 5 ml DEAE overnight at 4°C in a glass flask. It was then poured into a plastic column which was washed with 10 volumes column buffer and eluted via a step salt gradient consisting of 2 x 5 ml 200 mM NaCl, 2 x 5 ml 300 mM NaCl, 1 x 5 ml 500 mM NaCl (see Fig. 4*C*, column 2). Fractions were assayed by western blotting with antibody PMF-C13 and those fractions positive for p63 were pooled, concentrated and loaded onto preparative 2D gels.

**IsoElectricFocussing-PAGE 2D gels.** IEF was performed in an IPGPhor II unit (Amersham Biosciences, now GE Healthcare) using electrophoresis programs based on recommendations by the manufacturer; typically 18 hour runs over 60,000 V-hours total. Ascidian extract (total protein or partially purified) was loaded onto Immobiline DryStrip Gels (Amersham) in rehydration buffer containing 8M Urea. After isoelectric focusing, the gels were equilibrated with DTT and iodoacetamide and loaded onto the second dimension SDS-PAGE gels which were either stained for total protein or transferred to nitrocellulose for immunoblot.

To determine the isoelectric point of p63, IEF was initially performed on a series of drystrip gels spanning pH ranges between pH3 and pH11. Immunoblots with antibody PMF-C13 showed a clean signal around pI 5.2, and thereafter pH4-7 strips were used to achieve maximum separation in this region. In these initial analyses loaded with 750 μg total protein extract from *Phallusia* eggs or *Ciona* ovary, p63 was not abundant enough to be visible by Coomassie staining above the background of total protein content. Therefore for protein isolation and sequencing, a sample highly enriched for p63 was first prepared by sequential ion exchange chromatography as described above (“scaled-up prep”). This p63 enriched fraction was concentrated to 100 μl, mixed with 900 μl rehydration buffer and loaded onto two identical 24cm immobiline drystrip gels (pH range 4-7) which were run simultaneously in the multi well IPGPhor manifold (GE Healthcare). The central 16 cm were cut from the IEF strips and applied as stacks to two vertical protein gels. After migration one 2D gel was transferred for immunoblot using PMF-C13, and the twin gel was stained with Coomassie blue.

**Selection of spots on 2D gels corresponding to positive immunoblot signals.** It is not possible simply to overlay the western result onto the duplicate gel because the large second dimension gels are not perfectly identical and furthermore they change size during staining and transfer. In order to select the spot on the coomassie stained gel which corresponded to the positive signal on the western, the following point of reference system was used. A needle dipped in India Ink (Franco Maison, Nice France) was used to make pinpricks in the corners of the immunoblot after transfer but before the nitrocellulose was removed from the acrylamide. The transferred gel was then treated with the highly sensitive protein gel stain SyPro ruby (GE Healthcare), such that residual spots of the most abundant proteins were visible, giving a pattern that was easily recognizable in the second coomassie-stained gel. After western detection on Hyperfilm (GE Healthcare), the pinpricks allowed the physical overlay of the immunoblot result with the transferred gel. The target protein (p63) was located among the coomassie stained proteins by aligning the major spots between the two gels. Unambiguous selection was aided by the fact that unlike 1D bands, 2D protein spot signals display unique shapes (rarely perfect circles). The p63 spot (arrow in Fig. 4*D*) was excised, eluted from the acrylamide and sequenced by Proteomics Core Facility Biogenouest. The tryptic peptides were analyzed by Maldi-Tof yielding peptide mass fingerprint spectra, and certain peptides were sequenced by tandem MS/MS analysis.

**Preparation and labelling of cortices.** In this method, eggs or zygotes are affixed to polylysine coated coverslips and the cytoplasm is removed by a shearing force using a gentle stream of buffer X (350mM K-aspartate, 130mM taurine, 170mM betaine, 50mM glycine, 19mM MgCl_2_ and 10mM Hepes buffer). Here (Fig. 7), fertilized eggs were used for cortex preparation because we have noted that the retention of mitochondria is optimal in zygotes beginning to divide. Cortices were labeled with the lipophilic dye DiOC_6_(3) at 1 µg/µl, then fixed in freshly prepared 3.7% formaldehyde 0.1% glutaraldehyde in CIM buffer (800 mM glucose, 100 mM KCl, 2mM MgCl_2_, 5mM EGTA, 10mM MOPS buffer pH7.0), and immunolabelled with NN18 and PMF-C13 antibodies either directly or after membrane permeabilization with 0.1% Triton X 100 in CIM as in [3,4] (Sardet et al 1992; Sardet et al 2011).

1. Satou Y, Mineta K, Ogasawara M, Sasakura Y, Shoguchi E, et al. (2008) Improved genome assembly and evidence-based global gene model set for the chordate Ciona intestinalis: new insight into intron and operon populations. Genome Biol 9: R152. doi:10.1186/gb-2008-9-10-r152.

2. Endo T, Ueno K, Yonezawa K, Mineta K, Hotta K, et al. (2011) CIPRO 2.5: Ciona intestinalis protein database, a unique integrated repository of large-scale omics data, bioinformatic analyses and curated annotation, with user rating and reviewing functionality. Nucleic Acids Res 39: D807–814. doi:10.1093/nar/gkq1144.

3. Sardet C, Speksnijder J, Terasaki M, Chang P (1992) Polarity of the ascidian egg cortex before fertilization. Development 115: 221–237.

4. Sardet C, McDougall A, Yasuo H, Chenevert J, Pruliere G, et al. (2011) Embryological methods in ascidians: the Villefranche-sur-Mer protocols. Methods Mol Biol 770: 365–400. doi:10.1007/978-1-61779-210-6_14.
